# Prenatal Exposure to BPA Alters the Epigenome of the Rat Mammary Gland and Increases the Propensity to Neoplastic Development

**DOI:** 10.1371/journal.pone.0099800

**Published:** 2014-07-02

**Authors:** Eugen Dhimolea, Perinaaz R. Wadia, Tessa J. Murray, Matthew L. Settles, Jo D. Treitman, Carlos Sonnenschein, Toshi Shioda, Ana M. Soto

**Affiliations:** 1 Department of Anatomy and Cellular Biology, Tufts University School of Medicine, Boston, Massachusetts, United States of America; 2 Institute for Bioinformatics and Evolutionary Studies, University of Idaho, Moscow, Idaho, United States of America; 3 MGH Center for Cancer Research, Massachusetts General Hospital, Charlestown, Massachusetts, United States of America; Universidad Miguel Hernández de Elche, Spain

## Abstract

Exposure to environmental estrogens (xenoestrogens) may play a causal role in the increased breast cancer incidence which has been observed in Europe and the US over the last 50 years. The xenoestrogen bisphenol A (BPA) leaches from plastic food/beverage containers and dental materials. Fetal exposure to BPA induces preneoplastic and neoplastic lesions in the adult rat mammary gland. Previous results suggest that BPA acts through the estrogen receptors which are detected exclusively in the mesenchyme during the exposure period by directly altering gene expression, leading to alterations of the reciprocal interactions between mesenchyme and epithelium. This initiates a long sequence of altered morphogenetic events leading to neoplastic transformation. Additionally, BPA induces epigenetic changes in some tissues. To explore this mechanism in the mammary gland, Wistar-Furth rats were exposed subcutaneously via osmotic pumps to vehicle or 250 µg BPA/kg BW/day, a dose that induced ductal carcinomas *in situ*. Females exposed from gestational day 9 to postnatal day (PND) 1 were sacrificed at PND4, PND21 and at first estrus after PND50. Genomic DNA (gDNA) was isolated from the mammary tissue and immuno-precipitated using anti-5-methylcytosine antibodies. Detection and quantification of gDNA methylation status using the Nimblegen ChIP array revealed 7412 differentially methylated gDNA segments (out of 58207 segments), with the majority of changes occurring at PND21. Transcriptomal analysis revealed that the majority of gene expression differences between BPA- and vehicle-treated animals were observed later (PND50). BPA exposure resulted in higher levels of pro-activation histone H3K4 trimethylation at the transcriptional initiation site of the alpha-lactalbumin gene at PND4, concomitantly enhancing mRNA expression of this gene. These results show that fetal BPA exposure triggers changes in the postnatal and adult mammary gland epigenome and alters gene expression patterns. These events may contribute to the development of pre-neoplastic and neoplastic lesions that manifest during adulthood.

## Introduction

The link between the fetal exposure to environmental perturbations and the diseases manifested in adulthood is generally referred to as “developmental origins of adult disease” [1], [2]. Perturbations of the hormonal milieu of the embryo/fetus lead to dysfunction that manifests in adult life. Epidemiological data showed that high doses of pharmacological agents as well as increased prenatal levels of endogenous estrogens, such as those existing in twin pregnancies, may increase the susceptibility to breast cancer in adulthood [3], [4]. These facts provide the bases for the hypothesis that prenatal exposure to environmental estrogens induce malformations of the male genital tract, obesity, infertility [5] and the increased incidences of uterine leiomyoma, and testicular, prostatic and breast cancers observed in European and US populations over the last 50 years [6]–[9].

Among environmental hormonally active agents, the xenoestrogen bisphenol A (BPA) has received much attention due to widespread human exposure as suggested by the detection of BPA in the urine of 92% of a representative sample of the non-institutionalized U.S. population over 6 years of age [10], including pregnant women [11]. BPA can leach from certain plastic food/beverage containers [12], [13] dental materials [14] and epoxy resins. BPA has been reported to be present in plasma of men, women, fetuses and children as well as in the placenta [15], [16].

Gestational exposure of female rodents to BPA results in a constellation of signs that constitute the fetal xenoestrogen syndrome, which was first described in humans and rodents exposed to DES [17]. In rodents, the syndrome is characterized by early onset of puberty [18], early vaginal opening [19], disrupted estrus cyclicity [20], decreased luteinizing hormone levels following ovariectomy [21], decreased fertility and fecundity [22], and early cessation of ovarian cyclicity. In the fetal mouse mammary glands, exposure to BPA altered the composition of the stromal matrix, increased adipose maturation and ductal growth, delayed lumen formation and altered the pattern of gene expression in both the stroma and epithelium [23], [23]–[25]; comparable morphological changes were observed in the newborn primate [24]. At 4 months of age, the exposed animals showed a significant increase of lateral branches [26] and only animals exposed to BPA developed intraductal hyperplasias [27]. Also, mammary glands of BPA-exposed mice that were ovariectomized at prepubertal age showed an enhanced sensitivity to estradiol, demonstrated by an increase in the number of terminal end buds (TEBs), TEB area, TEB density and ductal extension [26]. Wistar Furth rats that were exposed *in utero* to 2.5, 25, 250 and 1000 µg BPA/kg body weight/day had significantly increased rates of ductal hyperplasia; additionally, those exposed to the two highest doses developed ductal carcinoma *in situ* at postnatal days (PND) 50 and 95 [28]. These highly proliferative lesions contained an increased number of cells expressing estrogen receptor alpha [28]. BPA binds and activates estrogen receptors alpha and beta [29]. During the period of exposure, i.e. prenatally, these estrogen receptors are detectable only in the primary mesenchyme. Because the propensity to develop mammary cancer manifests long after cessation of exposure, the plausible events following BPA exposure may include extemporaneous gene expression that underlies the altered stroma-epithelium interaction observed in the fetal mammary gland, as well as long-lasting changes in the epigenome.

Epigenetic changes are known to regulate gene expression and are thought to be involved in normal development and cancer [30], [31]. Maternal exposure to 20 µg BPA/kg/day induces genome-wide epigenetic alterations in the forebrains of mice at embryonic day (E) 12.5 and E14.5 [32]. Neonatal exposure of rats to 10 µg BPA/kg or to 0.1 µg estradiol/kg induced alterations in DNA methylation patterns of multiple genes in the prostate. BPA also increased the susceptibility of the prostate of these animals to adult-onset precancerous lesions following exposure to an additional carcinogenic stimulus (i.e., treatment with androgen and estrogen) [33]. Prenatal BPA exposure also leads to hypomethylation of the estrogen-responsive gene Hoxa10 in the uterus and concomitant perturbation of the developmental regulation of uterine estrogen response in mice [34].

Despite accumulated evidence linking BPA exposure to mammary carcinogenesis, the effect of fetal exposure to BPA on the mammary gland epigenome remains unexplored. In the present study, we have monitored BPA serum concentrations in dams in order to relate internal dose to the effects of BPA in the mammary gland. We also examined the effect of such prenatal exposure to BPA on the genome-wide DNA methylation status of the mammary gland during postnatal development and explored potential cues linking epigenetic alterations to breast carcinogenesis during adulthood. We conclude that *in utero* BPA exposure, at doses within the range of concentrations reported in bio-monitoring studies, is associated with genome-wide epigenetic changes and relevant transcriptional changes at all the time points studied, from the end of exposure (PND4) to adulthood, when intraductal hyperplasias and ductal carcinomas *in situ* (DCIS) are observed.

## Methods

### Animals

Sexually mature female Wistar-Furth rats (8-week-old; Harlan, Indianapolis, IN) were maintained in temperature- and light-controlled (14-h light, 10-h dark cycle) conditions at the Tufts University School of Medicine animal facility. All experimental procedures were approved by the Tufts University–Tufts Medical Center Animal Research Committee in accordance with the Guide for Care and Use of Laboratory Animals. Cages and bedding tested negligible for estrogenicity by the E-SCREEN assay; water was supplied from glass bottles only. Food (Harlan Teklad 2018) was supplied ad libitum. The E-SCREEN test indicated that the feed contained 20 femtomoles of estradiol equivalents per gram, which was considered a negligible amount. Female rats were mated with Wistar-Furth males of proven fertility and the morning on which sperm was observed in vaginal smears was designated embryonic day 1 (E1). On E9, the rats were weighed and implanted with Alzet osmotic pumps (DURECT Corp., Cupertino, CA) designed to deliver 250 µg BPA/kg body weight (BW)/day. The control animals were implanted with a pump delivering 50% dimethyl sulfoxide (vehicle control; Sigma Chemical Co., St. Louis, MO). The fetuses were exposed to BPA or vehicle from E9 to PND1. The litter was considered the unit of exposure; therefore only 1 female pup per litter was included in each experimental group. The animals were sacrificed at PND4 (6 vehicle- and 5 BPA-treated), PND21 (5 vehicle- and 5 BPA-treated) and at first estrous after PND50 (4 vehicle- and 4 BPA-treated) and their mammary gland tissue was stored at −80°C. The PND4 pups were sacrificed by decapitation and their sex was determined by anogenital distance and confirmed by the presence of ovaries and uteri. Rats of ages PND21 and PND50 were subjected to CO2 exsanguination followed by puncturing of the diaphragm.

### Quantification of circulating levels of BPA

At GD7, pregnant rats were weighed and this weight was used to calculate the BPA dose for each animal. BPA was delivered to the dam (and therefore the developing fetuses) via Alzet osmotic pumps loaded to deliver vehicle, 25, or 250 µg BPA/kg BW/day. Vehicle control animals were implanted with a pump delivering 50% dimethyl sulfoxide. Pumps were implanted on GD9. Blood was collected by cardiac puncture 72 hours later. All analyses of total and unconjugated serum BPA were conducted at the Centers for Disease Control and Prevention (CDC) as previously reported [35]. Briefly, serum was either treated with β-glucuronidase/sulfatase to quantify the concentration of total BPA (conjugated plus unconjugated), or processed without enzyme treatment to quantify the concentration of unconjugated BPA. Then, serum concentrations were measured using on-line solid phase extraction coupled to high performance liquid chromatography-isotope dilution tandem mass spectrometry. The limit of detection (LOD) was 0.3 ng/mL.

### Anti-5-methylcytosine antibody validation

The specificity of 5-methyl cytosine enrichment immune-precipitation assay was validated by simultaneously immuno-precipitating gDNA isolated from the mammary gland of an adult rat, using three anti-5-methylcytosine (5meC) mouse monoclonal antibodies: MAb-5Mecyt-100 (Diagenode, Belgium), Bi-MeCy-0100 (Eurogentec, USA) and 33D3 (Abcam, USA). A monoclonal antibody raised against an unrelated protein (CITED1) [36] was used as the negative control. The 5meC enrichment maps obtained after hybridization of immune-precipitation products with input gDNA were compared with regard to the specificity of all four antibodies (Figure S1 A). Microarray data analysis showed that the three anti-5meC antibodies had similar methylated gDNA enrichment profiles, as depicted by the methylation peaks, thus confirming the specificity of the assay. In contrast, no specific immuno-precipitation of methylated gDNA was observed with the control antibody. After comparing the performance of the three anti-5meC antibodies, Bi-MeCy-0100 was selected to immuno-precipitate gDNA fragments from the mammary glands of BPA- and vehicle-treated animals.

### Mathematical correction of microarray data bias

The heavily-methylated DNA segments tend to be GC-rich because methylation occurs only in the CpG dinucleotides, which are found mostly in the CpG islands. Because the GC-rich DNA segments hybridize better than GC-poor segments, the two-color hybridization used in the methylation microarrays shows discordance at the region of low signal intensities and the signal for the methylated DNA diminishes significantly in the low-intensity range. The divergence between the regression curves of the real signal (microarray readout, red line) and the theoretical signal values (readout as if the signal was only dependent on immuno-precipitation-efficiency, blue line) are represented as the M-A plots in Figure S1B left. The Roche Nimblegen commercial microarray data analysis software does not take into account the fact that GC-rich fragments by default will give stronger microarray signal than GC-poor segments. We applied an algorithm that calculates the thermodynamic differences among different microarray probes using their GC contents and corrects the hybridization results, i.e. the microarray signals for GC-rich probes were decreased and GC-poor signals were enhanced appropriately (Figure S1B) [37].

### Chromatin immunoprecipitation

Genomic DNA (gDNA) was isolated from the whole mammary glands using the QIAamp kit (Qiagen, Germantown, MD) according to the manufacturer's instructions. The isolated gDNA was enzymatically fragmented by 1 h incubation at 37°C in the presence of the digestion enzyme MseI. Digested gDNA (1 µg) was stored for further use (input gDNA); the remaining amount was immunoprecipitated by overnight incubation with anti-5meC antibodies (10 µg/ml IgG final concentration) at 4°C under rotation, in order to enrich for methylated gDNA (IP gDNA). Three antibodies were compared for their specificity and efficiency to immunoprecipitate methylated gDNA, namely: MAb-5Mecyt-100 (Diagenode), Bi-MeCy-0100 (Eurogentec) and 33D3 (Abcam), while an anti-CITED1 [36] antibody was used as negative control. The antibody-gDNA complex was incubated with Protein A agarose beads (Invitrogen) under rotation for 2 h at room temperature, followed by three subsequent centrifugation/washing cycles and overnight incubation with Proteinase K (0.4 mAU/ml final concentration) at 55°C. Next, the immunoprecipitated gDNA was extracted using phenol and precipitated by the standard ethanol/LiCl method. The effects of BPA on H3K4 methylation was similarly tested by using the Active Motif anti-histone H3K4me3 rabbit polyclonal antibody (ChIP grade, cat# 39159) with PCR primers amplifying a 88 nt gDNA fragment spanning the *Lalba* transcription initiation site (forward primer 5′-GCGAAATACACGCCAGGAAC, reverse primer 5′-GCAGGTGAAGTGAGTGGGAT).

### Genomic DNA analysis

In order to obtain sufficient material for microarray hybridization, 10 ng of IP and input gDNA from each sample were amplified using the GenomePlex Complete Whole Genome Amplification 2 (WGA2) kit (Sigma, St. Louis, MO). The amplification product was purified using the QIAquick PCR Purification kit (Qiagen) and 50 ng of WGA2-amplified gDNA were re-amplified using the WGA3 kit (Sigma, USA) in order to generate >10 µg gDNA for each sample. The WGA3-amplified IP and input gDNA was purified again using the QIAquick kit, quantified and subsequently labeled with the Cy5 and Cy3 dyes respectively. The labeled samples were hybridized to the ChiP-chip RefSeq Promoter array for rat (RN34 RefSeq promoter, NimbleGen, Reykjavik, Iceland). Signal intensity data was extracted by scanning images of each array using NimbleScan and visualized using the software Signal Map (NimbleGen).

### Genomic DNA microarray data analysis

BPA-treated and control samples were compared with regard to array signal intensity and only differences with p values <0.05 were considered. The enrichment p-values for each probe were generated by NimbleGen using a proprietary statistical method; peaks (methylation-enriched spots) were subsequently generated based on the p-values. GC-rich immuno-precipitated fragments tend to hybridize better with the probes, compared to GC-poor regions, introducing a bias on the readings in the region of low signal intensities. An algorithm that calculates the thermodynamic differences among different microarray probes using their GC contents was applied in order to minimize the discordance between theoretical and real values and mathematically correct the hybridization results. After identifying the differences in methylation profile between BPA-treated and control animals, UCSC Genome Browser was used to identify the genes in the vicinity of methylation changes.

### Gene expression microarrays

Total RNA from the mammary glands of animals treated with BPA or vehicle was isolated using the RNeasy Lipid Tissue kit (Qiagen Inc., Valencia CA), which uses a combination of Qiazol followed by column extraction. Next, 50 ng of the isolated RNA was used to generate amplified cDNA using the WT-Ovation Pico RNA Amplification System (NuGEN, San Carlos, CA). Five µg of amplified cDNA from each sample were labeled with biotin using the Encore Biotin Module kit (NuGEN) and subsequently hybridized to the Rat Genome 230 2.0 Chip Arrays (Affymetrix). Eight microarray data sets, which represented 4 BPA-treated and 4 control animals, were obtained for each time point (24 microarrays in total). Raw data were generated by the GeneChip Scanner 3000 using the appropriate software. Pairwise analysis between the two treatment groups was performed using the GeneSifter.net on-line service (vizXlabs, Seattle, WA) and gene expression differences with p<0.05 were identified.

### Real-time PCR

Two micrograms of total RNA was treated with DNase I (Invitrogen/Life Technologies, Grand Island, NY), according to the manufacturer's protocol, to remove residual gDNA. This RNA sample was reversed transcribed with Superscript III First-strand cDNA Synthesis Kit (Invitrogen) in the presence of oligo(dT) (Invitrogen), as per the manufacturer's instructions. Two µl of the resulting cDNA was mixed with SYBR-Green Supermix (Bio-Rad) and 0.4 µM of each primer in a 25-µl PCR reaction and thermocycled in the Bio-Rad iQ5 real time PCR detection system.

## Results

### Choosing the BPA dose

The internal BPA dose was measured in the serum of pregnant Wistar/Furth rat dams exposed to 0, 25 and 250 µg BPA/kg BW/day at 72 hours following pump implantation. Total BPA (conjugated plus unconjugated) was detected in 67% of the 25 µg BPA/kg BW/day animals and in 100% of the 250 µg BPA/kg BW/day animals (Table 1). Unconjugated BPA was undetectable in dams exposed to 25 µg BPA/kg BW/day but was detected in all dams exposed to 250 µg BPA/kg BW/day (1.68±0.74 ng/ml). Hence, animals used in the epigenome and transcriptome studies were exposed to 250 µg BPA/kg BW/day, which unlike the lower dose induced not only hyperplasias but also DCIS.

**Table 1:** Serum BPA mean concentrations (± standard deviation) in dams at GD12, 72 hours after Alzet pump implantation on GD9 (LOD: limit of detection = 0.3 µg/L; LOQ: limit of quantification = 0.9 µg/L.)

BPA dose (µg/kg/d)	Total BPA (µg/L)		% detectable	Unconjugated BPA (µg/L)		% detectable
0 BPA	***<LOD***	N = 5	0% (0/5)	N/A	N = 0	N/A
25 BPA	***0.72±0.60 (all animals)*** † 1.02±0.46 n = 4 (only detectable levels)	N = 6	67% (4/6)	***<LOD***	N = 4	0/4
250 BPA	9.76±3.85	N = 5	100% (5/5)	1.68±0.74	N = 5	100% (5/5)

† Instrument generated values were used for this calculation even if they were below the LOD.

### BPA exposure *in utero* altered epigenetic regulation during post-natal mammary gland development

**Figure 1:**
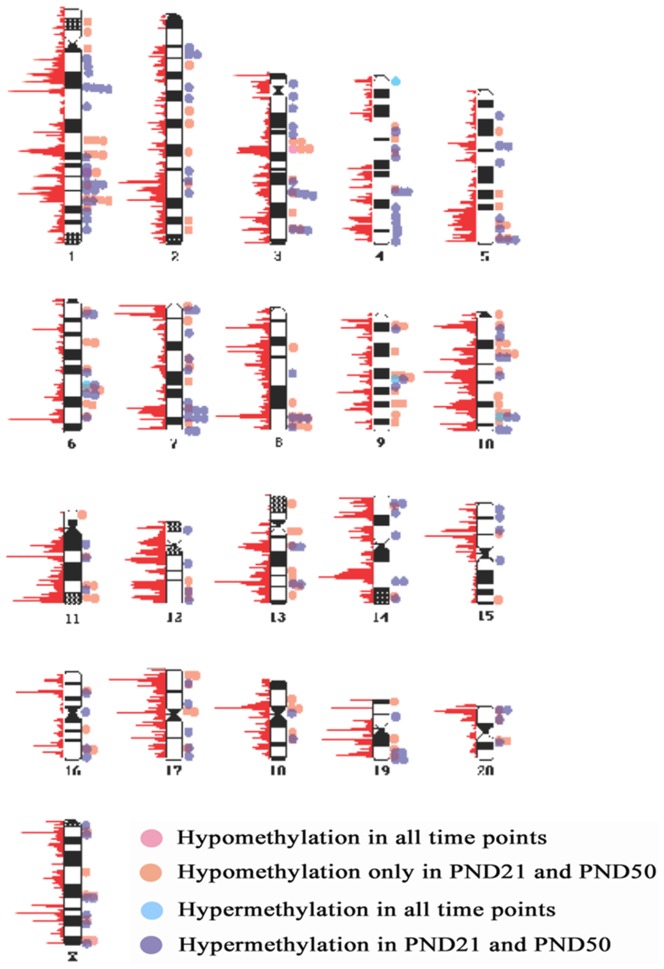
Karyogram of BPA-exposed rat mammary glands indicating altered methylation patterns. BPA-induced changes in DNA methylation at PND4, PND21 and PND50. The red graphs to the left of each chromosome indicates relative numbers of the transcriptional initiation sites found within a 1 megabase window hence, corresponding to the density of promoters.

The experimental design is schematically represented in Figure S2. The control and BPA-treated groups represent 4 to 6 litters each. Only statistically significant changes are considered. Animals born from BPA-treated mothers displayed changes in methylation status in multiple genomic loci when compared to offspring of vehicle-treated mothers. Because the DNA methylation analysis was performed using microarrays covering only the rat RefSeq gene promoters, the BPA-related changes detected in our study were distributed unevenly on the chromosomes but enriched in the regions with high gene densities (Figure 1). The observed distribution of the affected DNA methylation suggests the widespread nature of the epigenetic effects of BPA throughout the chromosomes. The gDNA methylation changes that were identified did not show any significant trend toward hypo- or hyper-methylation at any of the tested time-points. Of the 58207 genomic loci probed in total after in utero BPA exposure, 812 were hypermethylated and 675 were hypomethylated at PND 4, 1904 were hypermethylated and 1787 hypomethylated at PND 21, and 1072 were hypomethylated and 1162 hypomethylated at PND 50. The highest number of methylation changes was observed at the prepubertal stage (PND21) (Figure S3).

The methylation patterns induced by BPA showed dynamic changes over time. Few gDNA methylation differences between BPA- and vehicle-treated groups were maintained throughout the three time-points. Only 41 probed chromosomal segments (out of 1904) that were hyper-methylated at PND 4 remained in the same state at PND 21, and only 5 of these sites remained hyper-methylated at PND50. Conversely, 38 probed segments (out of 675) that were hypo-methylated at PND4 remained hypo-methylated at PND21, and only 2 of those segments were hypo-methylated at PND50. Hypo- and hyper-methylation were defined as significant signal differences between BPA- and vehicle-treated groups at any given time point (t-test, p<0.05). Thus, loss of hypo-methylation or hyper-methylation of a specific chromosomal segment between two time-points in the BPA-treated group would not necessarily imply a change in the number of methylated CpG dinucleotides, but may reflect instead a time-dependent methylation change in the vehicle-treated group that did not occur in the BPA-treated group.

### BPA exposure *in utero* altered gene expression during post-natal mammary gland development

**Figure 2:**
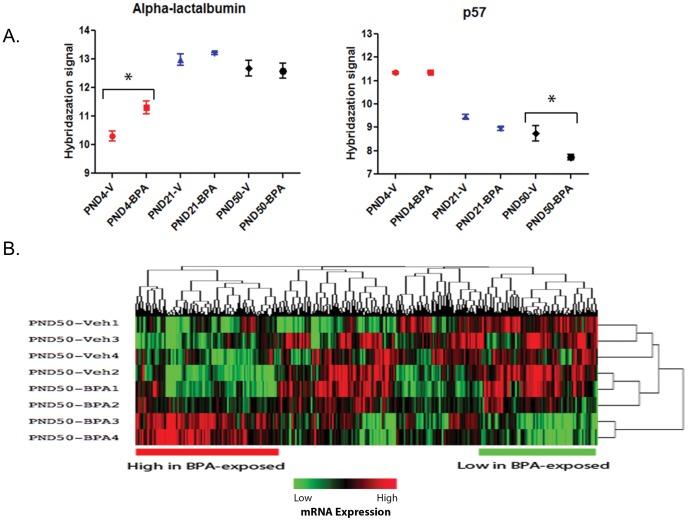
Alterations in gene expression in mammary glands following BPA-exposure. **A.** Up-regulation of alpha-lactalbumin gene expression at PND4 (left), and down-regulation of *p57* gene expression at PND50 (right) in the BPA-treated group. **B.** Heat map representing gene expression changes between BPA- and vehicle-treated animals at PND50.

**Figure 3:**
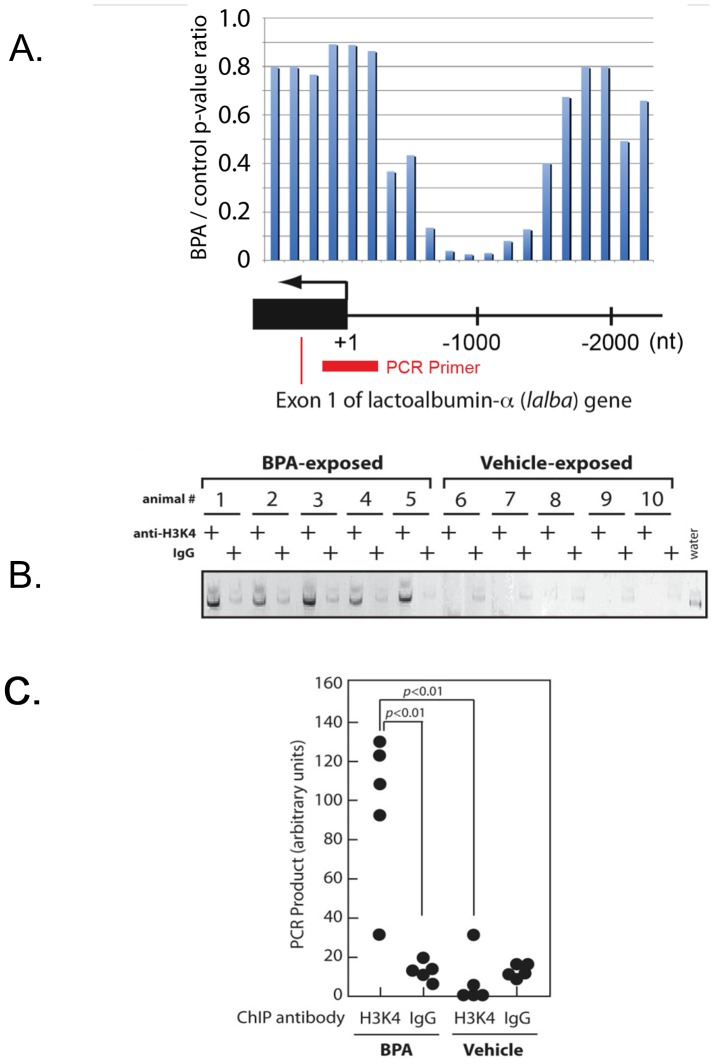
Altered epigenetic markers of the alpha-lactalbumin (*Lalba*) gene observed in BPA-exposed mammary glands. A. BPA-induced DNA demethylation near the *Lalba* promoter. p-values of the DNA methylation calculated from the microarray-based DNA methylation analysis are indicated. The PCR amplicon position for the ChIP-seq assay of H3K4me3 is shown as a red bar. B. ChIP assay of H3K4me3 at the *Lalba* transcription initiation site in the PND4 mammary glands. C. Quantitation following histone immunoprecipitation indicates enrichment of the *Lalba* promoter for the histone H3K4.

Transcriptomal analysis did not show widespread changes in mRNA expression in the mammary gland between BPA- and vehicle-treated groups at PNDs 4 and 21. Nonetheless, BPA-exposure induced approximately a 2-fold increase in the expression of alpha-lactalbumin gene at PND 4 (Figure 2A). However, there was no difference in alpha-lactalbumin expression at PNDs 21 and 50 between BPA- and vehicle-treated groups. Paradoxically, the promoter region of alpha-lactalbumin gene on chromosome 7 did not show any changes in methylation status between the two groups at PNDs 4 and 21, but was significantly hypo-methylated at PND 50 suggesting a gDNA methylation-independent regulation mechanism for this gene (Figure 3A).

Large-scale changes in the mRNA expression profile induced by BPA exposure were first observed at PND 50 (Figure 2B), concomitant with the appearance of intraductal hyperplasias and DCIS. The BPA-treated group showed increased expression of *jun* and down-regulation of the *p57* gene (also known as *cdkn1c*) both known to be involved in cell cycle regulation (Figure 2A). A list of genes with significant gene expression changes is shown in Table S1.

### In-utero BPA exposure altered histone H3K4 methylation at the alpha-lactalbumin promoter

Because exposure to BPA increased expression of the alpha-lactalbumin mRNA transcript at PND4 (Figure 2), we attempted to determine whether this enhanced transcription is associated with trimethylation of histone H3 lysine 4 (H3K4me3), which is an established epigenetic marker of actively transcribed genes [38]. Chromatin from the mammary gland samples was immuno-precipitated using an antibody against H3K4me3 and subjected to PCR amplification of a 88-bp genomic DNA region spanning the transcription initiation site of the alpha-lactalbumin gene. This ChIP assay revealed significant enrichment of H3K4me3 at the alpha-lactalbumin promoter in BPA-treateed mammary glands compared to the control (Figure 3B). This suggests the possibility that the enhanced alpha-lactalbumin gene expression at PND4 in the BPA-exposued mammary gland involves epigenetic levels of regulation.

## Discussion

In rodents, exposure to BPA during embryonic development increases susceptibility to mammary carcinogenesis during adulthood which manifests as intraductal hyperplasias and DCIS [28] and palpable carcinoma tumors [39]. When these animals are exposed to a second carcinogenic stimulus at puberty or adulthood they develop palpable carcinomas [40]–[42]. Ho and collaborators postulated that alterations in the DNA methylation patterns of multiple genes identified in the prostate of BPA-exposed rats may be the underlying cause of neoplastic development of this organ later in life [33]. In contrast, most investigators in the field of cancer research have supported for almost one century the notion that cancer is due to the accumulation of mutations in a cell (somatic mutation theory) [43]; this notion does not apply to BPA, a non-mutagenic chemical [9], [44]. Both the mutational and epigenetic theories of carcinogenesis imply that cancer originates in one cell that has undergone genetic and/or epigenetic changes, which ultimately result in dysregulated cell proliferation [45]. Alternatively, the tissue organization field theory (TOFT) postulates that carcinogenesis represents a problem of tissue organization, comparable to organogenesis gone awry, and that proliferation is the default state of all cells [46], [47]. According to this theory, carcinogens and teratogens disrupt the normal dynamic interaction of neighboring cells and tissues during early development and/or adulthood [48] which is consistent with the theory of fetal origins of adult diseases [1], [2].

In the case of BPA, disruptions of epithelial- mesenchymal interactions may be due to a direct effect of the xenoestrogen on regulation of gene expression in the mammary mesenchyme, the only compartment expressing estrogen receptor in the fetal mammary gland. Indeed, changes in the ECM and the presumptive fat pad were accompanied by changes in the expression of key genes coding for ECM component, adipogenesis, and stromal-epithelial interactions [25]. Here we present evidence showing that *in utero* BPA exposure induces changes in the epigenetic profile in the rat mammary gland that manifest at all time-points examined after cessation of exposure. However, genome-wide changes in mRNA expression were only observed at PND50, the time at which increased incidence of intraductal hyperplasias and DCIS are observed. Epigenetic changes including DNA methylation and chromatin modifications have been proposed as plausible explanations for these effects of BPA [49].

In the present study, pregnant rats were exposed to 250 µg/kg BPA daily from E9 to birth, a dose that results in detectable blood serum concentrations of unconjugated BPA in the mother as well as in the fetus at gestational day 21 [39]. Fetal exposure to this, environmentally relevant [50], BPA dose resulted in intraductal hyperplasias and carcinoma *in situ* in adult rats [28]. Intraductal hyperplasias are considered to be precursors of malignant tumors in both rats and humans [51] and, most mammary carcinomas in rats and humans are estrogen-dependent. Indeed, lesions formed in the adult rat mammary gland after *in utero* exposure to BPA have high expression of ER-alpha suggesting that these tumors are also driven by estrogens [28]. In this regard, prenatal exposure to BPA enhances the response of the mammary glands to ovarian estrogens during puberty and adulthood [26], probably contributing to the carcinogenic process. Indeed, puberty is considered a window of high susceptibility to chemical carcinogens in rats [52], [53] similar to irradiation for humans [54].

BPA-induced alterations in the DNA methylation profile were observed throughout the genome at all three time points. Differences in methylation patterns consisted of both hypo- and hyper-methylation events which were distributed throughout the genome (Figure 1). This observation suggests that the effect of BPA is not limited to particular areas in the genome. The highest number of the methylation differences was observed at PND 21, a time when estrogens are secreted by the ovary, 13 days before the opening of the vagina and puberty. This developmental period is associated with major tissue remodeling in the mammary gland which are characterized by epigenetic events including changes in DNA methylation and chromatin modifications [55]. The large-scale differences in the DNA methylation profile between the two experimental groups may be a consequence of the alterations observed in the fetal mammary gland during BPA exposure [25].

While limited BPA-induced changes in gene expression occurred at PND21, large scale differences in transcriptomal profiles between BPA- and vehicle-treated animals were observed at PND50, a period characterized by adult levels of estrogen and ovarian cyclicity. These differences between vehicle and BPA-exposed rats may be due to the fact that fetal BPA exposure enhances the response to estrogens [26] and progesterone [56]. The appearance of these lesions at puberty is reminiscent of the timing of manifestation of diethylstilbestrol-induced clear cell carcinoma of the vagina in women, suggesting that exposure to ovarian hormones contribute to the development of these pathologies [57]. The gene expression changes at PND 50 reported herein, simultaneous with the manifestation of ductal hyperplastic lesions and DCIS, are likely to represent the molecular level manifestation of the neoplasias induced by BPA, which span the molecular, cellular and tissue levels of organization. In this regard, it is not surprising that genes associated with increased cell proliferation (such as *jun*, *cdkn1c* etc.) are found up-regulated at this time point.

We could find limited evidence linking changes in methylation status to changes in gene expression. For instance, of the 43 genes differentially regulated at PND 50 only 13 had methylation changes within 2 kbp distance from the respective transcription initiation sites. Additional epigenetic mechanisms that are not related to CpG methylation could also be involved in gene expression regulation. For example, the enrichment of the *lalba* promoter for H3K4me3 indicates that this gene could be regulated by histone methylation-dependent mechanism at this time point. The scale of epigenetic effects caused by *in utero* BPA exposure other than CpG methylation and their impact on mammary gland development remain to be investigated.

In conclusion, these results show that fetal exposure to BPA induces large scale epigenetic changes in the rat mammary gland throughout adult life. The role of these time-dependent methylation alterations remains unclear. However, these changes appear to reflect the developmental aberrations caused by BPA rather than representing the causal events that result in carcinogenesis later in adulthood, which seem to involve altered stromal-epithelial interactions in the fetal mammary gland.

## Supporting Information

Figure S1 **Optimization of antibodies and algorithms used to analyze altered DNA methylation patterns.** **A.** Comparison of gDNA enrichment in various chromosome regions using three anti-5meC antibodies (MAb-5Mecyt-100, Bi-MeCy-0100 and 33D3) and a negative control antibody: CITED-1; notice the similar peak distribution for the anti-MeCy antibodies. **B.** Computational correction of two-color Cy3/Cy5 microarray fluorescence signal bias based on local GC contents. Distribution of gDNA methylation microarray signals before (left) and after (right) correcting for interference by hybridization preference due to differences in GC content between probes.
(TIF)

Figure S2 **Schematic representation of the experimental design.**
(TIF)

Figure S3 **Altered DNA methylation in mammary glands following BPA-exposure.** Schematic representation of chromosomal areas with methylation status differences between BPA- and vehicle-treated groups throughout the examined time-points. Red indicates change in methylation status.
(TIF)

Table S1 Genes with significant gene expression changes.
(DOC)
