# Supplementary figures and images for: Prenatal Exposure to BPA Alters the Epigenome of the Rat Mammary Gland and Increases the Propensity to Neoplastic Development
Source: PLoS One. 2014 Jul 2;9(7):e99800. doi: 10.1371/journal.pone.0099800 (PMC4079328; doi:10.1371/journal.pone.0099800)

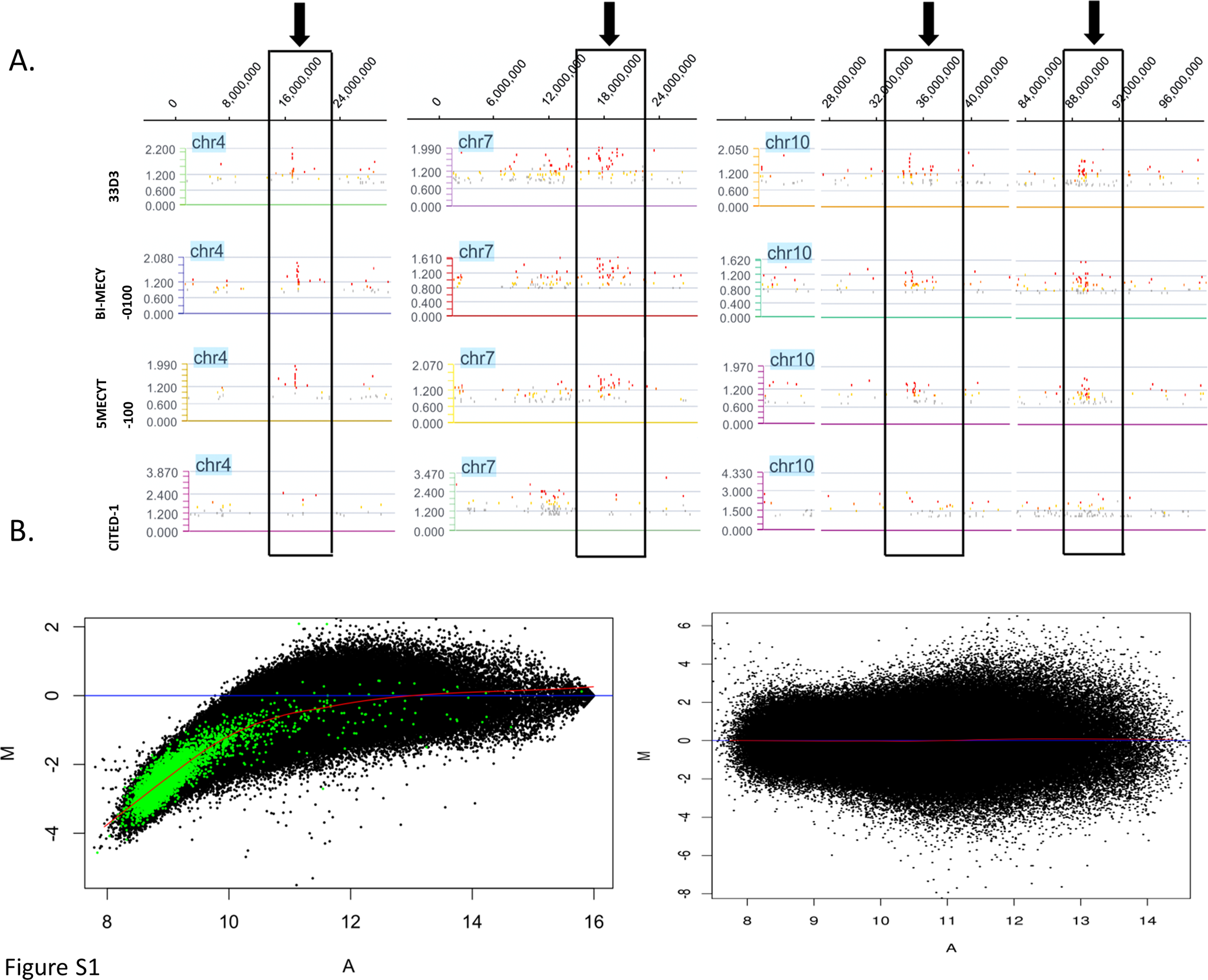

Supplement: Figure S1 — Optimization of antibodies and algorithms used to analyze altered DNA methylation patterns. A. Comparison of gDNA enrichment in various chromosome regions using three anti-5meC antibodies (MAb-5Mecyt-100, Bi-MeCy-0100 and 33D3) and a negative control antibody: CITED-1; notice the similar peak distribution for the anti-MeCy antibodies. B. Computational correction of two-color Cy3/Cy5 microarray fluorescence signal bias based on local GC contents. Distribution of gDNA methylation microarray signals before (left) and after (right) correcting for interference by hybridization preference due to differences in GC content between probes. (TIF) [file pone.0099800.s001.tif]

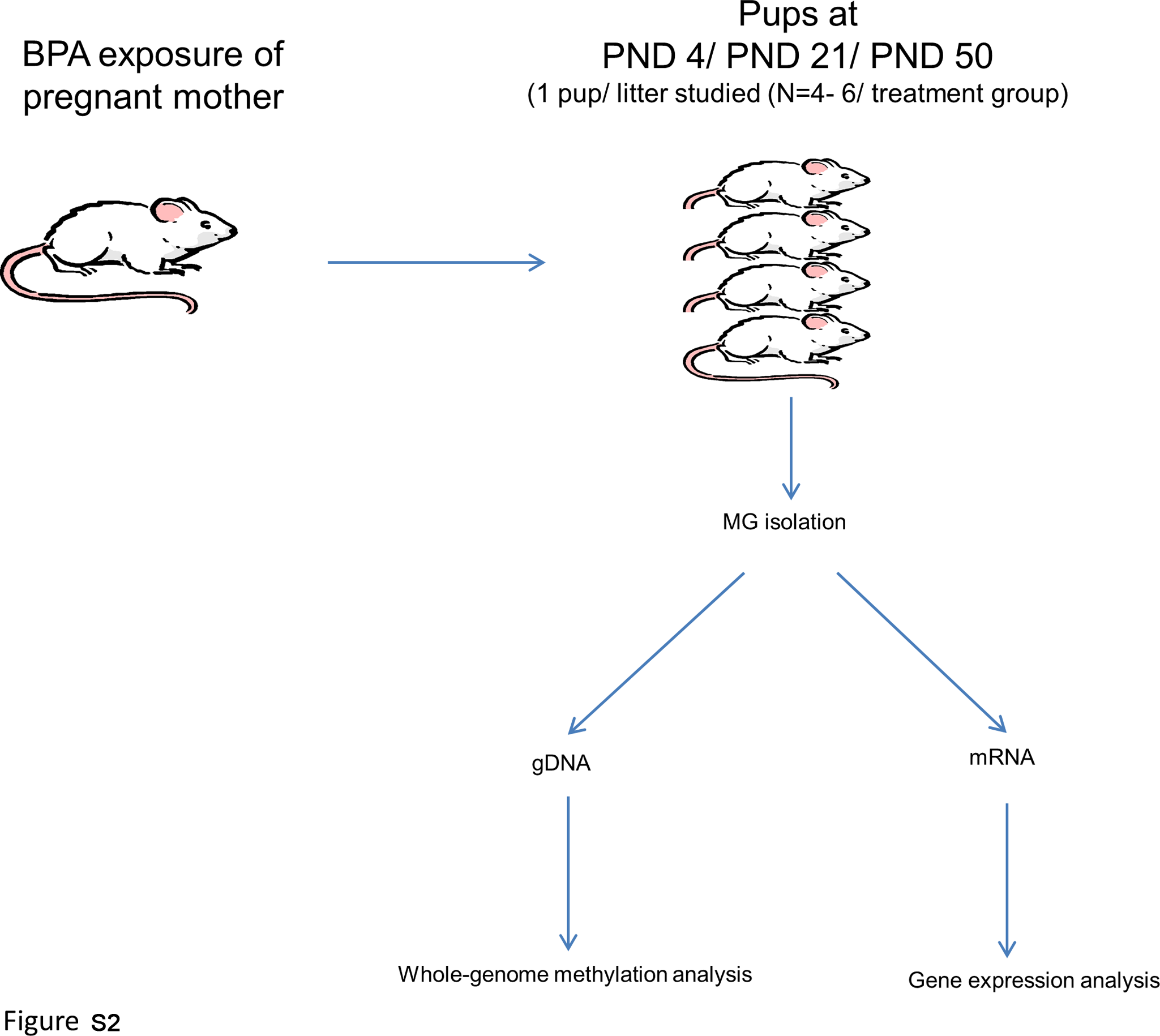

Supplement: Figure S2 — Schematic representation of the experimental design. (TIF) [file pone.0099800.s002.tif]

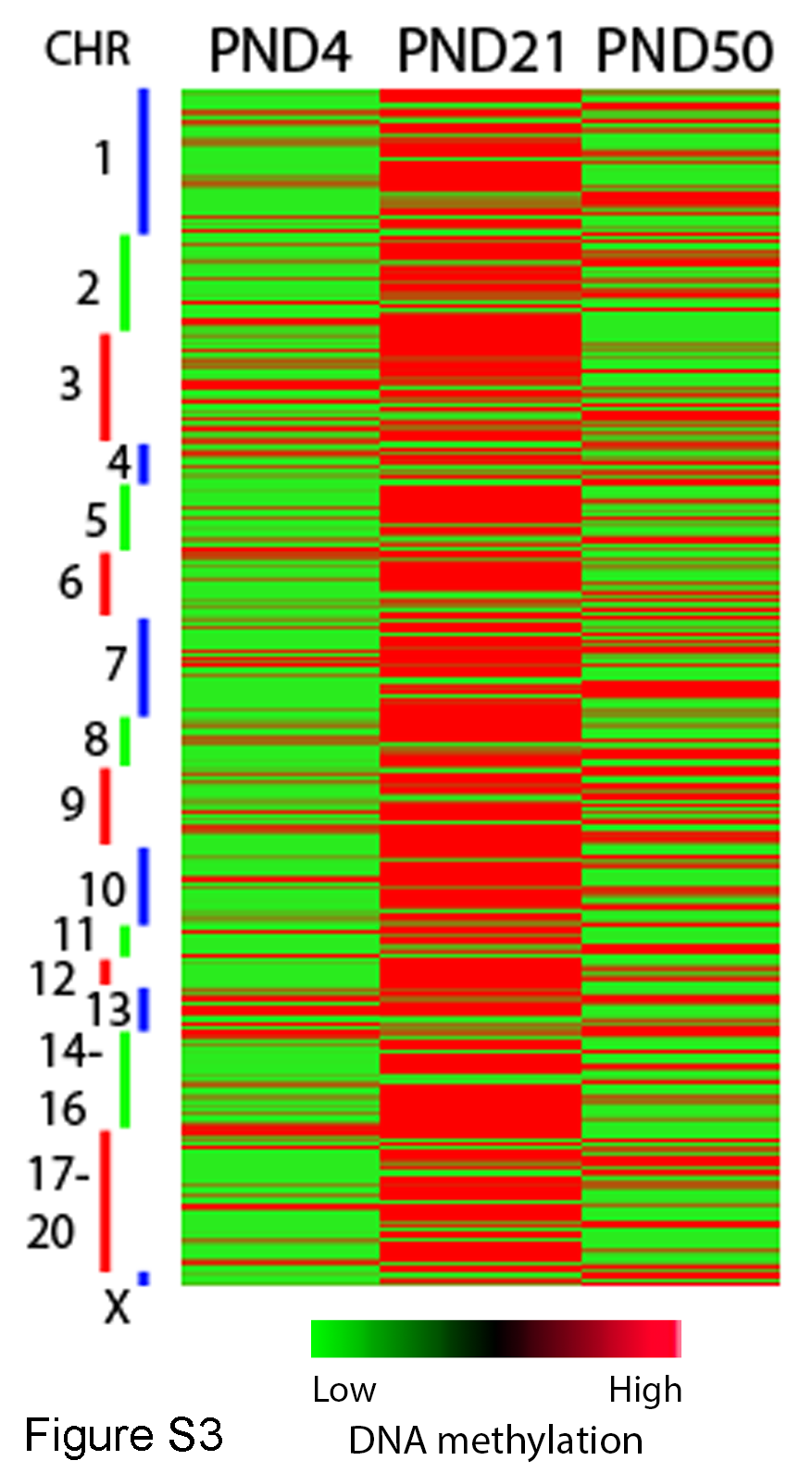

Supplement: Figure S3 — Altered DNA methylation in mammary glands following BPA-exposure. Schematic representation of chromosomal areas with methylation status differences between BPA- and vehicle-treated groups throughout the examined time-points. Red indicates change in methylation status. (TIF) [file pone.0099800.s003.tif]
