# Supplementary material for: Prenatal Exposure to BPA Alters the Epigenome of the Rat Mammary Gland and Increases the Propensity to Neoplastic Development
Source: PLoS One. 2014 Jul 2;9(7):e99800. doi: 10.1371/journal.pone.0099800 (PMC4079328; doi:10.1371/journal.pone.0099800)
Supplement: Table S1 — Genes with significant gene expression changes. (DOC) [file pone.0099800.s004.doc]

| Ratio | Direction | p-value | Gene Name |
| --- | --- | --- | --- |
| 2.48 | **↓** | 0.01036 | Fibrinogen gamma chain |
| 2.43 | **↓** | 0.028681 | Transcribed locus |
| 2.37 | **↓** | 0.040311 | Neuronal PAS domain protein 2 |
| 2.19 | **↓** | 0.019831 | Cyclin-dependent kinase inhibitor 1C (P57) |
| 1.9 | **↓** | 0.014575 | Amyloid beta (A4) precursor protein-binding, family B, member 1 interacting protein |
| 1.74 | **↓** | 0.027376 | Family with sequence similarity 110, member B |
| 1.71 | **↓** | 0.031544 | Chemokine (C-C motif) ligand 5 |
| 1.57 | **↓** | 0.043682 | Inhibitor of DNA binding 2 |
| 1.54 | **↓** | 0.040243 | Transcribed locus, strongly similar to NP_758478.1 coiled-coil and C2 domain containing 2A [Mus musculus] |
| 2.54 | **↑** | 0.023572 | Flavin containing monooxygenase 2 |
| 2.41 | **↑** | 0.027532 | Transcribed locus |
| 2.33 | **↑** | 0.032189 | Solute carrier family 2 (facilitated glucose transporter), member 4 |
| 2.32 | **↑** | 0.009308 | Similar to tyrosine kinase-associated leucine zipper protein LAZipII |
| 2.27 | **↑** | 0.046348 | Acetoacetyl-CoA synthetase |
| 2.23 | **↑** | 0.033494 | Acetyl-coenzyme A carboxylase alpha |
| 2.14 | **↑** | 0.042352 | Transcribed locus |
| 2.07 | **↑** | 0.034732 | Solute carrier family 25 (mitochondrial carrier, citrate transporter), member 1 |
| 2.06 | **↑** | 0.04451 | Cullin 2 |
| 2.03 | **↑** | 0.025984 | Transcribed locus |
| 2.02 | **↑** | 0.039495 | Tetratricopeptide repeat domain 25 |
| 1.96 | **↑** | 0.022671 | Transcribed locus |
| 1.96 | **↑** | 0.042288 | Zinc finger, DHHC-type containing 8 |
| 1.95 | **↑** | 0.047129 | FK506 binding protein 5 |
| 1.91 | **↑** | 0.007319 | Pyruvate dehydrogenase phosphatase isoenzyme 2 |
| 1.88 | **↑** | 0.037109 | Secretory carrier membrane protein 2 |
| 1.83 | **↑** | 0.033935 | Selenoprotein W, 1 |
| 1.78 | **↑** | 0.043096 | Wee 1 homolog (S. pombe) |
| 1.77 | **↑** | 0.012808 | Transcribed locus |
| 1.76 | **↑** | 0.045215 | Jun oncogene |
| 1.76 | **↑** | 0.041678 | TSC22 domain family, member 3 |
| 1.74 | **↑** | 0.043087 | Hemoglobin alpha, adult chain 2 |
| 1.72 | **↑** | 0.030338 | Diacylglycerol O-acyltransferase homolog 2 (mouse) |
| 1.63 | **↑** | 0.04108 | Transcribed locus |
| 1.61 | **↑** | 0.015766 | Hypothetical protein LOC681219 |
| 1.6 | **↑** | 0.043581 | Kelch domain containing 3 |
| 1.59 | **↑** | 0.045794 | Glycosyltransferase 25 domain containing 1 |
| 1.54 | **↑** | 0.020453 | PDZ domain containing 8 |
| 1.53 | **↑** | 0.042884 | Glutamate-ammonia ligase (glutamine synthetase) |
| 1.52 | **↑** | 0.046448 | Transcribed locus |
| 1.47 | **↑** | 0.048338 | Mitochondrial ribosomal protein L45 |
| 1.43 | **↑** | 0.045955 | Beta globin minor gene |
| 1.43 | **↑** | 0.045209 | Calmodulin 3 |
| 1.3 | **↑** | 0.042275 | --- |
